# Measuring the gradualist approach to internationalization: Empirical evidence from the wine sector

**DOI:** 10.1371/journal.pone.0196804

**Published:** 2018-05-04

**Authors:** Mónica Clavel San Emeterio, Rubén Fernández-Ortiz, Jesús Arteaga-Ortiz, Pablo Dorta-González

**Affiliations:** 1 Economics and Business Department, University of La Rioja, Logroño, Spain; 2 Business and Management Department, University of Las Palmas de Gran Canaria, Las Palmas de Gran Canaria, Spain; Universidad de Castilla-La Mancha, SPAIN

## Abstract

The objective of this paper is to fill a gap in the literature on internationalization, in relation to the absence of objective and measurable performance indicators for the process of how firms sequentially enter external markets. To that end, this research develops a quantitative tool for use as a performance indicator of gradualness for firms entering external markets at a sectoral level. The performance indicator is based on firms’ export volumes, number of years operating in the export market, geographic areas targeted for export and when exports began to each area. The indicator is tested empirically in the wine sector. The main contribution of this study is the creation of a reliable international priority index, which can serve more widely as a valuable tool because of its potential use in other industry sectors and geographic areas, and which would allow the analysis of how geographically differentiated internationalization strategies develop.

## Introduction

The study of foreign market entry modes has essentially focused on factors related to the concept of psychic distance (PD) [1, 2]. Numerous factors that influence the attributes of PD have been identified and explain how they determine, at least partially, location choices and the sequence of events leading to entry by firms into new, foreign markets [3]. However, there are no studies in the literature which characterize this pattern of behavior. The question, which remains unanswered, is how are sequencing decisions made when an industry or sector moves into external markets? In order to answer this question, we do not analyze the influencing factors but, rather, the steps by which an industry enters an external market.

In answering this question, this paper fills a research gap by formulating a tool for measuring, independent of any sector, gradualness of external market entry. We propose an objective indicator which, by using market entry date entry sequence and the commitment to undertake export activities, will create a measurement of the gradualness process in the internationalization of businesses. We test the tool empirically by applying it to the Spanish wine industry, analyzing the results of their historic decisions to enter foreign markets. The empirical test validates how this objective indicator is generalizable to other industries in measuring the gradualness pattern of market entry. The indicator will allow us to generalize from the Spanish wine sector through the examination of its gradualness approach to opening foreign markets.

The paper is organized as follows. Section 2 provides the theoretical grounding for the analysis through a review of the theoretical bases which underpin external market entry mode theory. Next, we identify the different applications of these concepts in the theory and research on International Business (IB). In Section 3 we formulate a set of metrics to measure the steps taken to enter external markets. Section 4 describes the gradualist approach to internationalization. In Sections 5 and 6 we test and verify the validity of the proposed international priority index (IPI) through its application to a key Spanish industry—wine production—both from an economic and an export perspective. In section 7, we present the main conclusions about the robustness, validity and appropriateness of the proposed IPI, in addition to its implications for research methodology in IB. In the final section, limitations of the study and directions for future research are discussed.

## Literature review

The gradualist approach, or Uppsala model, is considered the best-known and most important in the study of the internationalization of small and medium sized enterprises [4, 5, 6]. The strategy of internationalization is defined as a gradual process, based on the seminal study by Johanson and Wiedersheim-Paul [7] published in 1975. According to this perspective, when a firm develops an internationalization strategy, given its lack of experience and regular information, it will start with sporadic export activities to psychically proximate destinations; thereafter, it will incrementally advance both in the use of its resources and in its international implications, as its international experience increases [6, 8, 9]. In this body of work, it is argued that companies begin their internationalization processes in countries with closer psychic distances (PD) before venturing to more psychically distant countries [6, 8, 10]. As argued by Johanson and Vahlne [11], entering countries that are psychically close reduces the level of market uncertainty. According to Kogut and Singh [12], it is easier for these firms to learn about markets in countries that are psychically close, because there is an implicit assumption that psychically close countries are more similar and similarity is easier for firms to manage than dissimilarity, thereby making it more likely that they will succeed [10]. Since psychic distance affects companies’ perceptions of uncertainty and, as this is a cognitive measure based on human perceptions and what they know, or believe they know, managers will make the decision to internationalize based not only on the uncertainty they feel about the new foreign market but also based on their internal uncertainty about their ability to operate in the new market [13].

Thus, a firm's first steps will be toward psychically closer markets since they can be considered extensions of the domestic market, requiring only small adjustments in their operations, systems and processes [14, 15]. However, this gradual, evolutionary process has been criticized [5, 16, 17, 18, 19, 20, 21, 22] and has not been empirically tested [23]. This is due to, among other reasons, the difficulty researchers have had in defining an objective, quantitative and measurable indicator that synthesizes and correctly differentiates methods of opening external markets. Works such as Luo et al. [24], Pangarkar [25] and Sahaym and Nam [26] have used indicators like export commitment (external sales over total sales), or export volume, to measure export intensity. Other research [26] has measured export experience (number of years exporting), number of foreign customer relationships [27], destination of exports or PD to study sequencing processes in internationalization. PD, has been, without doubt, the measure most widely used in the numerous works [28] which have tried to model market entry processes. However, the most recent challenge in psychological distance takes place in the operationalization of the concept [13]. Therefore, next we discuss how international business (IB) literature has addressed PD.

As noted by Zaheer et al. [2], international management is the management of distance [29]. The PD concept was introduced by Beckerman [30] to explain the perceived differences (subjective) between home and host countries that affect internationalization. According to Joliet and Hubner [31], PD can be defined as the perceived distance between the home country and a foreign country, resulting in cultural, business and political differences, i.e. differences in language, political and legal systems, trade practices, industry structure, etc.

The PD concept has been widely used in IB literature [29] to explain how firms internationalize in terms of market selectivity and how they develop knowledge about foreign markets [1, 2, 3, 7, 12, 13, 29, 31, 32, 33, 34, 35, 36, 37, 38, 39, 40, 41, 42, 43, 44]. Research into IB has considered PD as a central element in the understanding and explaining of internationalization phenomena and usually considers it an obstacle to internationalization [37]. Ambos and Hakanson [45] observed that 29% of the 285 studies undertaken on PD from 1975 to 2011 related to market selection, while 25% centered on entry mode related outcomes. The need for globalization in destination markets suggests that PD is likely to have a greater impact on SMEs’ operations after foreign market entry than it has on market selection decisions [44]. Similarly, as noted by Cho and Padmanabhan [46], no study on internationalization can be complete unless it has an explicit variable that controls PD [29].

To operationalize PD, the following indicators have been used: level of economic development, education levels, business language, culture and local languages, business practices, political systems, per capita incomes, cultural values, lifestyles, traditions [47, 48, 49], geographical distance, cultural distance, market influx, market size and a questionnaire of managers’ perceptions about the distance between countries. However, as stated by Langhoff [50], in this conception of PD it is not possible to conclude that the internationalization process depends on a company's knowledge, but rather it depends on information in the public domain. From a methodological point of view, the validity of this approach to measuring PD (exogenous index) has been criticized [2, 29], and researchers are now beginning to use an individual level measurement [47] to better assess how the perception of PD affects business decisions and behavior [29].

Researchers habitually refer to cultural distance and psychic distance without explaining the difference between the two concepts [48, 29]. Barkema et al. [32], Benito and Gripsrud [33], Kogut and Singh [12], and Padmanabhan and Cho [42] align the meaning of PD more closely to the notion of cultural distance, based on Hofstede’s [51] 1980 work, using cultural distance almost as a synonym and proxy for PD [10]. Kogut and Singh’s [12] work has been used in numerous IB studies [29].

Following this same line of thought, Ronen and Shenkar [52], based on a review of empirical works which used Hofstede's index, identified various cultural groups by calculating the difference between the index of the cultural group in a company’s home country, and different foreign cultural groups, and obtained a measurement of the cultural distance between different groups of countries and export zones.

Kostova [39] introduces the concept of institutional distance as a means to capture, on a broad front, the differences between countries, including not only cultural differences, but also regulatory and normative distance [29].

Finally, Clark and Pugh [53] measured PD through a construct formed by four independent variables. These were defined as “Market size”, “Market influx”, “Geographical distance” and “Cultural distance”. More recently, following the same line of thought, other authors, like Martin and Drogendijk [1], have proposed PD measures typically based on publicly available statistics and studies on cultural values, creating a construct formed by cultural distance, physical distance and socioeconomic distance.

Other authors, like Klein and Roth [38] and Nordström [40], among others, have developed a version of the PD concept from answers that managers have given to questions on the difficulty of entering foreign markets. This line of thought continues to present difficulties for the globalization and homogeneity of the PD variable. Authors, such as O´Grady and Lane [10], argue that PD should also encompass industry structure and the competitive environment. As noted by Ambos and Hakanson [45], the concept of PD doubtless owes much of its attractiveness to its inherent vagueness. Our understanding of the impact of PD has long been limited by flawed conceptualizations and unreliable measures.

Although there is a lot of literature on the subject, the net effect of PD is still represented as an unresolved problem in the IB literature [29]. There is an open debate about the symmetry or asymmetry of the concept, about the phenomenon at the individual, microeconomic or macroeconomic level and about the objective or perceptive indicators of the measurement [29, 37]. Thus, many authors remain unclear about the exact understanding of the concept and the underlying reasons for the operationalization employed [37].

As we have observed, when operationalizing the gradualness construct, an important and significant discordance is found in the literature. The study of PD, or its operationalization, has been criticized [2, 43, 54, 55, 56, 57, 58, 59, 60, 61, 62, 63] and it does not fulfill the needs that, from a research point of view, must be met to identify a measure of the gradualist process of internationalization. IB researchers are making efforts to discover the cognitive mechanisms that form distance perception [29]. Thus, if an internationalization pattern is found for a productive area (defining what countries a firm targets and when internationalization occurs), it must take into account that each of the companies under study will be directing their activities to different areas and using different timescales. They acquire higher levels of international commitment as they accumulate experience and knowledge. This knowledge will also help reduce their levels of uncertainty [64].

We conclude, therefore, that the empirical literature on IB needs an indicator that reflects the behavior of real businesses and foreign market patterns and that will allow the quantification, for each individual company, of how proximate or distant they are from the pattern of behavior established by the structure of the industry [10]. In the next section, we propose an objective indicator that, by using market entry date, entry sequence and commitment to exporting, will create a measurement of the gradualness process in the internationalization of businesses.

## Materials and methods

Since there is no quantitative measure that indicates if a specific firm, or sector, follows the internationalization gradualist model, we propose the creation of an internationalization priority index that will measure the degree to which firms follow the export behavior outlined in the gradualist methodology literature. The need for this index is supported by the earlier research of Davidson [65] and Clark and Pugh [53].

We consider three main factors in the export process: the order of entry into the export zones followed by each firm; the export width (number of total years since entry) of the firms in each zone; and export depth (amount of exported product) of the firms in each zone.

Based on Davidson [65], a comparison by pairs of zones (dyads) in the order followed by each exporting firm allows us to establish a ranking of international priority. Later, this ranking will be weighted by the width and depth of the exports.

For each zone dyad, all the firms that have simultaneously carried out international operations in the two zones are examined, assigning a value of 1 if the firm directed its activities first to the zone whose index we are calculating (zone *z*) and 0 in the opposite case. In this way, we obtain the number of firms that entered zone *z* before entering the rest of the zones examined. This provides an international priority measure for zone *z*. This procedure can be followed for each zone, obtaining an international priority ranking for each zone.

The export entry year of a firm to each zone provides a measure of the export width. We define the export width of firm *f* in zone *z* in the following way:

$$ExportWidth_{z}^{f}=\frac{ExportYears_{z}^{f}}{TotalExportYears^{f}},$$ (1)

where *ExportYears*^*f*^_*z*_ is the number of years that firm *f* has been exporting to zone *z* and *TotalExportYears*^*f*^ is the total number of years that firm *f* has been exporting to any zone. As we are exclusively studying firms involved in exports, the denominator of this ratio is always greater than zero.

This ratio belongs to the interval [0.1] and it is a proportion of the number of years exporting to each zone. For example, *ExportWidth*^*f*^_*z*_ *= 0*.*6* means that firm *f* has been exporting for 60% of its total export years to zone *z*. In specific cases, a value of 1 means exports to zone *z* began in the first year, and a value of 0 means that there have been no exports to zone *z*.

The proportion of exports to each zone provides a measure of the depth of exports. We define the export depth of firm *f* in zone *z* in the following way:

$$ExportDepth_{z}^{f}=\frac{ExportVolume_{z}^{f}}{TotalExportVolume^{f}},$$ (2)

where *ExportVolume*^*f*^_*z*_ is the amount that firm *f* exports to zone *z* and *TotalExportVolume*^*f*^ is the total amount that firm *f* exports to all zones.

This ratio belongs to interval [0.1] and is a proportion of the exports to each zone. For example, *ExportDepth*^*f*^_*z*_ = 0.4 means that firm *f* sends 40% of its exports to zone *z*.

Once we have considered all the variables in the export process (the export order, the export width, and the export depth), we define the sectoral *International Priority Index (IPI)* of an export zone *z* as follows:

$$IPI_{z}=\sum_{\begin{array}{c} i=1\\ i\neq z \end{array}}^{n}\sum_{f\in F}(ExportWidth_{z}^{f}∙ExportDepth_{z}^{f}),$$ (3)

where *n* is the total number of export zones and *F* is the set of firms that select first *z* then *i*.

This measure can be normalized in intervals [0.1] by dividing by *max*_*z = 1*,*2*,*…n*_ *(IPI*_*z)*,_ to facilitate its interpretation as a priority rate. Therefore, we define the sectoral *Normalized International Priority Index (NIPI)* of an export zone *z* as following:

$$NIPI_{z}=\frac{IPI_{z}}{max_{z=1,2,\ldots .,n}\left\{IPI_{z}\right\}},$$ (4)

As an example, *NIPI*_*z*_ *= 0*.*85* means that zone *z* has a priority of 85%.

Once the different priority indexes are obtained, we can determine which zones have a greater priority for a given firm or sector. Thus, if we rank the *IPI* obtained for the different zones under consideration, we can establish the order of international priority (sectoral order) in the destination of activities abroad.

In order to clarify the calculation of the Normalized International Priority Index (NIPI), we present below an example with four companies internationalizing in four different zones (A, B, C and D).

The first IPI calculated is Zone A, which we compare in dyads with the other three zones. For that purpose, we pick the first dyad (Zone A–Zone B) and observe (see Table 1) how the four companies developed their international activities in both zones; only Company 1 and Company 3 entered Zone A before entering Zone B. Thus, using the calculated data on depth and width for Zone A (see Table 2), we add the product of depth and width of the two companies who entered Zone A before Zone B.

**Table 1:** Start of the companies’ internationalization processes for each of the zones.

	*Year of internationalization*				
*Firms*	*Zone A*	*Zone B*	*Zone C*	*Zone D*	*Total Export Years*
*Firm 1*	1990	2000	1985	-	28
*Firm 2*	2001	1997	-	2005	16
*Firm 3*	1986	2001	1993	1980	33
*Firm 4*	2005	2003	1994	-	19

**Table 2:** Depth and width for each of the companies in the different zones.

	*Zone A*		*Zone B*		*Zone C*		*Zone D*	
*Firms*	*Depth*	*Width*	*Depth*	*Width*	*Depth*	*Width*	*Depth*	*Width*
*Firm 1*	0.30	0.82	0.20	0.46	0.50	1.00	-	-
*Firm 2*	0.20	0.75	0.40	1.00	-	-	0.40	0.50
*Firm 3*	0.10	0.82	0.40	0.36	0.20	0.61	0.30	1.00
*Firm 4*	0.50	0.42	0.30	0.53	0.20	1.00	-	-

We move now to the next dyad, Zone A–Zone C. In this dyad, three companies have been directing their export activities to both zones (Company 1, Company 3 and Company 4), and we observe (see Table 1) how only Company 3 entered Zone A before entering Zone C, so we take the product of depth and width from Company 3 in Zone A (see Table 2).

Finally, we compare Zones A and D, where we find that only Company 2 and Company 3 exported to both zones. Of these two, Company 2 exported first to Zone A, and Company 3 exported first to Zone D, so we take the product of depth and width of Company 2 in Zone A.

Once the results for the dyadic comparisons of Zone A are obtained, we aggregate them to obtain the IPI value for Zone A (IPI Zone A). To calculate the IPI of the remaining zones we proceed in the same manner, obtaining the results shown in Table 3.

**Table 3:** Calculation of IPI for the four zones.

*Zones*	*Zone Dyads*	*IPI disaggregated*	*IPI Total*
***Zone A***	A—B	0.33	0.56
	A—C	0.08	
	A—D	0.15	
***Zone B***	B—A	0.56	0.96
	B—C	0.00	
	B—D	0.40	
***Zone C***	C—A	0.70	1.52
	C—B	0.82	
	C—D	0.00	
***Zone D***	D—A	0.30	0.90
	D—B	0.30	
	D—C	0.30	

Once the IPIs of the different zones in the example are calculated, to normalize the index we divide each of them by the maximum value reached in all zones. In the proposed example, it would be the value reached by the IPI in Zone C (1.52), so the different NIPI values for this example would be as shown in Table 4.

**Table 4:** NIPI and sectoral order.

	*ZoneA*	*ZoneB*	*ZoneC*	*ZoneD*
***IPI***	0.56	0.96	1.52	0.90
***NIPI***	0.37	0.63	1.00	0.59
***NIPI x 100%***	37%	63%	100%	59%
***Sectoral order***	4	2	1	3

Once the normalized international priority for the zones used in this example are calculated, and the order of internationalization in the activity sector of the four fictitious companies is considered, the NIPI, in order from high to low, would be as seen in Table 4.

The interpretation of the NIPI x 100% row in Table 4 is as follows. For the analyzed sector, the highest priority zone is Zone C, with a 37% (100–63) higher priority than Zone B, a 41% (100–59) higher priority than Zone D and a 63% (100–37) higher priority than Zone A. In addition, Zone B has a 4% (63–59) higher priority than zone D, and so on.

### Sample and variables

The population under study is the Spanish wine sector, due to its economic importance for the country and its global significance. Spain currently has 0.98 million hectares of grape production, which makes it the country with the largest area of vineyards in the world, followed by China and France [66]. It is also the leading global exporter of wine by volume. To obtain the population under study (Spanish wineries), we used the SABI database (Iberian Balance Analysis System). This is a directory with contact data and financial information on more than 1,080,000 Spanish companies, classified by CNAE code (National Classification of Economic Activities). This gave us 2760 Spanish companies assigned to the CNAE code 11.02: wine-making process, which formed our population under study.

The measuring instrument used was a questionnaire, consisting of 18 items. Table 5 shows the variables considered in the study. We attempted to survey key informants in all the listed wineries (2760 wineries). The respondents were senior export managers. The survey was first conducted by email and later supplemented by a telephone survey. The questions were identical in both surveys. The number of valid questionnaires returned was 255, which is a response rate of 9.23%. This sample has a confidence level of 95%, so we can consider it adequate for this study. Of the population under study, 2760 Spanish wineries, 640 are exporters. 72.5% of the sample obtained are exporters. These data show a positive bias in our study, because we focus on the set of exporting wineries. If we take the total of Spanish wineries the overall response rate was 9.23%, rising to 28.9% of the exporting wineries.

**Table 5:** Variables introduced in the study.

*VARIABLE*	*ESTIMATOR*
Year of creation	Age
Starting year of international activities	Total export years
Year of entrance to EU	Export years EU
Year of entrance to USA	Export years USA
Year of entrance to rest of Europe	Export years rest EU
Year of entrance to Mercosur	Export years Mercosur
Year of entrance to rest of Latin America	Export years rest LA
Year of entrance to Asia	Export years Asia
Year of entrance to Australia	Export years Australia
Year of entrance to others	Export years others
Percentage of exports to EU	Export depth EU
Percentage of exports to USA	Export depth USA
Percentage of exports to rest of EU	Export depth rest EU
Percentage of exports to Mercosur	Export depth Mercosur
Percentage of exports to rest of Latin America	Export depth rest LA
Percentage of exports to Asia	Export depth Asia
Percentage of exports to Australia	Export depth Australia
Percentage of exports to others	Export depth others

Finally, in order to justify the reliability of this sample and eliminate the potential non-response bias, we applied the test suggested by Armstrong and Overton [67]. In order to do this, we performed a variance analysis between questionnaire answers obtained on two different occasions, obtaining a p-value above 0.05. This determined the non-existence of significant differences in the items of the two groups of questionnaires, confirming that the data obtained in our study does not present non-response bias, or bias due to conditioned response as a consequence of the data gathering method used.

The zones considered in the study were: European Union; Rest of Europe, U.S.A. and Canada; Mercosur, Rest of Latin America, Asia, Australia; and Other Destinations. The selection of these regions is justified as they are the main export markets for Spanish wine [68].

## Results, discussion and conclusions

### Results and discussion

The Spanish wine sector, in addition to its importance to the country's external image, has great importance for the economic value it generates (1% of Spanish GDP), for the population it employs and for the role it performs in environmental conservation [69].

The sector has shown a sustained recovery trend, which may principally be attributed to the export levels attained during the years under study. In 2016, Spanish consumption of wine is estimated at 1,000 million liters. Spanish wine exports, in 2016, were almost 2,226 million liters, that is, more than double domestic consumption. Therefore, it seems clear that it is the export sector that has most evolved in recent years.

Regarding international markets, results are encouraging and, as pointed out by a report published by the Spanish Wine Federation [70], Spain has become a net wine exporter. This means that the market growth is outside Spain’s borders.

Concerning the destination of Spanish wines in international markets, data show that Spanish wineries sell primarily in the European Union (64.79% of the exporting wineries) and American (52.57% of the exporting wineries) markets, leaving Asia (37.94% of the exporting wineries) and Oceania (7.60% of the exporting wineries) far behind.

We can now analyze the international priority order of the wine sector’s external market opening strategy. By using the normalized international priority index (NIPI) described in the previous section, we will be able to determine the strategic pattern used by Spanish wineries as they moved into external markets.

As mentioned above, for this study, the following regions were considered: European Union, Rest of Europe, U.S.A. and Canada, Mercosur, Rest of Latin America, Asia, Australia, and Other destinations. The geographic dimension is generally measured by the number of countries to which a firm exports [71], but following Rugman and Verbeke [72], we argue that where the international scope of a firm is concerned, regions rather than countries are the relevant units of analysis. The selection of the eight zones is based on an adaptation of the geographic divisions used by Vaaler and McNamara [73] and Cerrato and Piva [74] and on their identification as the main export zones for Spanish wine [68].

Table 6 shows some central tendency indicators and variable indicators required for the development of NIPI, as well as the age of the company and its exporting experience, differentiated by zone.

**Table 6:** Central tendency and variable measures by export zones.

*Zone*	*Variables*	*Width*	*Depth*	*Export Experience*	*Age*
***EU***	Mean	0.98	0.64	16.5	30.7
	S.D	0.11	0.31	17.1	28.4
***Rest of Europe***	Mean	0.87	0.18	16.0	27.6
	S.D	0.21	0.20	9.2	28.0
***USA and Canada***	Mean	0.85	0.30	16.8	28.1
	S.D	0.23	0.25	17.7	27.5
***Mercosur***	Mean	0.70	0.92	24.5	32.8
	S.D	0.26	0.98	29.3	32.8
***Rest of LA***	Mean	0.81	0.17	22.5	30.6
	S.D	0.24	0.19	29.3	30.1
***Asia***	Mean	0.79	0.18	15.1	25.8
	S.D	0.25	0.20	9.6	24.9
***Australia***	Mean	0.71	0.06	21.2	26.6
	S.D	0.29	0.06	11.8	13.1
***Others***	Mean	0.86	0.30	15.0	30.6
	S.D	0.31	0.32	8.7	20.6

The results obtained show that the entry order into the different zones was: European Union, U.S.A. and Canada, Rest of Europe, Other destinations, Asia, Rest of Latin America, Mercosur, and Australia. Additionally, the EU has a 69% higher priority than U.S.A. and Canada region, which has a 23% higher priority than the Rest of Europe, the Rest of Europe has a 1% higher priority than Other destinations, etc. (see Table 7)

**Table 7:** NIPI of the Spanish wine sector.

*Zone*	*IPI*	*NIPI*	*NIPI x 100%*	*Order*
***EU***	64.08	1.00	100%	1
***Rest of Europe***	5.36	0.08	8%	3
***USA and Canada***	19.59	0.31	31%	2
***Mercosur***	0.41	0.01	1%	7
***Rest of LA***	2.85	0.04	4%	6
***Asia***	4.11	0.06	6%	5
***Australia***	0.00	0.00	0%	8
***Others***	4.42	0.07	7%	4

These results show that, according to the Uppsala theory, companies initially direct their international activities toward nearby countries, in which PD is lower and, as they acquire experience, they start exporting to countries that are more psychically distant.

### Conclusions and implications

The literature on entry to external markets has focused almost exclusively on the study of PD, identifying the factors that have an impact on the concept. This article discusses several research works that have studied these factors, their composition and how they determine, at least partially, the destinations and the sequence in the opening of these external markets. However, the indicators used to measure this concept have been criticized [2, 43, 54, 55, 56, 57, 58, 59, 60, 61, 62, 63]. Some of the studies have focused on macroeconomic factors such as cultural distance, geographical distance, level of economic development, level of education; other studies have focused on microeconomic factors such as the perception of distance that managers have of different countries. Thus, despite there being a large body of literature that demonstrates that psychological distance has an impact on the pattern of entry into foreign markets, there is no single measurement of the concept, and few works have addressed patterns in the opening of external markets. Therefore, our first academic contribution is the review of the fragmented and limited literature in this field.

Also, according to the Uppsala model or gradualist theory, the opening of foreign markets is conditioned by psychological distance. The first steps of a company according to this model will be toward psychically closer markets, since they can be considered extensions of the internal market and involve less need to adjust operations, systems and processes [14, 15]. However, this gradual and evolutionary characterization has been criticized [5, 16, 17, 18, 19, 20, 21, 22] and is not empirically supported [23]. Among other reasons, this is due to the difficulties encountered by researchers when they try to establish an objective, quantitative and measurable indicator, with the capacity to synthesize and correctly discriminate methods of opening external markets. Based on this, this paper proposes a way to measure, for any sector, gradual entry into external markets by use of an indicator that implicitly incorporates psychological distance from the host country to other countries.

As we can extract from the previous paragraphs, there are numerous macroeconomic and microeconomic factors that make up strategies for entering foreign markets. The first conclusion and contribution of our work is the synthetic capacity of the indicator. Our index synthesizes these variables in an objective, metric indicator, making it comparable and compatible with different sectors while taking into account the time variable.

The second contribution is the creation of a quantitative methodology that can be used as an indicator of gradual performance for companies entering external markets at a sectoral level. In addition, by using a quantitative, measurable, objective and continuous indicator, this tool can be used as a variable dependent on export behavior patterns, or as an independent variable in studies that, for example, define strategies for the opening of external markets. In this way, we can analyze if success in the international marketplace is influenced by the speed of the process. This objective indicator, the IPI (International Priority Index), of the gradual entry into external markets at sector level, was developed based on export volume, years exporting, geographic export destination zones and start time of exports to each region. This indicator, as an inter-sectorial extrapolated measure, can be applied to any sector and will help us analyze how internationalization strategies are developed for different geographical areas, measuring the degree to which companies follow the export behavior pattern described in the gradualist methodology literature.

Another contribution of this work is that the International Priority Index is a measure of internationalization activity. This economic indicator allows the analysis of business performance and the prediction of future returns. Our indicator is factual—without distortions by opinion, personal feelings or prejudices—(objective), measurable, precise and consistent, so that data collected from different sources over time are not biased due to different sources or different periods (well defined). In addition, the indicator has a direct relationship with the internationalization process (valid) and is easy to obtain. Therefore, we consider that this tool is a contribution to IB and an important attempt to help its development. The index could have high utility for national, public institutions, as it can aid them to develop and adapt their policies of promotion and development of international activities in function of the behavior of the IPI.

Finally, the tool was tested empirically by applying it to the Spanish wine industry, analyzing the results of its historic decisions to enter foreign markets and validating the generalizable nature of this objective indicator. Having this indicator allows us to generalize from the Spanish wine sector by studying the export pattern of its gradual opening of foreign markets. As for Spanish wineries, our findings show that, in accordance with the Uppsala theory, companies initially direct their international activities to neighboring countries where PD is less and, as they gain experience, they export to other, more psychically distant countries.

From the perspective of the entrepreneur and economic agents, this indicator can simplify strategies for the opening of external markets, allowing them to define the attractiveness of different countries or regions for different economic sectors. This is, therefore, a third implication, in this case, for decision makers. Similarly, for the Spanish wine sector, this indicator can be employed to compare itself with other sectors, to analyze if others develop their export strategies in the same or in less gradual ways and if there are differences in how others achieve success abroad.

## Limitations and future research directions

The limitations of the study should be considered when the results are interpreted. Firstly, although the empirical data focused on a sample of Spanish wineries, the findings could be of interest to firms in other countries. However, readers should exercise caution in attempting to generalize this study’s findings to other, considerably different economic settings.

Regarding future research directions; taking into account that the main contribution of this study is the creation of an international priority index to serve as a valuable and reliable tool, because of its potential use in other industry sectors and geographic areas, allowing us to analyze how geographically differentiated internationalization strategies develop, it would be of value to replicate the study in different geographical contexts so that the results could be generalized to larger populations.

Secondly, this study focuses on a cross sectional research design performed at a given moment in time, with enterprises operating in different stages of the export process, and with different length of export experience, thus, no longitudinal analysis was performed. Future studies should consider employing longitudinal analyses in order to illustrate the dynamics of exporting. Thirdly, it may also be advisable to carry out similar studies with other industries, as well as to differentiate the results according to the relevant overseas markets.

Finally, this tool can be used in further studies as a variable dependent on export behavior patterns or as an independent variable. Thus, future lines of research could focus on an analysis of whether the companies known as “born global”, despite going out into the international market at an early stage after their inception, do so gradually or not.

Despite these limitations, and although the results need to be confirmed by further research, the study does provide preliminary answers to the research goals.

## Supporting information

S1 File Data set. (SAV)
